# Cerebrospinal Fluid Anti-Neuronal Autoantibodies in COVID-19-Associated Limbic Encephalitis with Acute Cerebellar Ataxia and Myoclonus Syndrome: Case Report and Literature Review

**DOI:** 10.3390/diagnostics13122055

**Published:** 2023-06-14

**Authors:** Konstantina Yiannopoulou, Aigli G. Vakrakou, Aikaterini Anastasiou, Georgia Nikolopoulou, Athina Sourdi, John S. Tzartos, Constantinos Kilidireas, Antonios Dimitrakopoulos

**Affiliations:** 1 Second Neurological Department, Henry Dunant Hospital Center, 115 26 Athens, Greece; k.giannopoulou.14@hotmail.com (K.Y.); geonikolopoulou@yahoo.gr (G.N.); kildrcost@med.uoa.gr (C.K.); 2 1st Department of Neurology, Eginition Hospital, Medical School, National and Kapodistrian University of Athens, Vas. Sofias 72-74, 11528 Athens, Greece; 3 REHAB Basel, Clinic for Neurorehabilitation and Paraplegiology, 4055 Basel, Switzerland; aikatianast@gmail.com; 4 Third Department of Internal Medicine, Henry Dunant Hospital Center, 115 26 Athens, Greece; sourdiath53@gmail.com (A.S.); a.dimitrakopoulos@dunant.gr (A.D.); 5 Second Department of Neurology, “Attikon” University Hospital, School of Medicine, National and Kapodistrian University of Athens, 124 62 Athens, Greece; jtzartos@gmail.com

**Keywords:** SARS-CoV-2, post-infectious, movement disorders, myoclonus, acute cerebellar ataxia, confusion, hallucinations, indirect immunofluorescence, plasmapheresis

## Abstract

Since the outbreak of coronavirus (COVID-19) in 2019, various rare movement disorders and cognitive changes have been recognized as potential neurological complications. The early treatment of some of these allows rapid recovery; therefore, we must diagnose these manifestations in a timely way. We describe the case of a 76-year-old man infected with severe acute respiratory syndrome coronavirus-2 who presented with confusion and hallucinations and was admitted to our hospital 14 days after the onset of symptoms. One day later, he developed generalized myoclonus, dysarthria and ataxia, and tonic clonic seizures and was admitted to the intensive care unit. A diagnosis of COVID-19-associated autoimmune encephalitis with characteristics of limbic encephalitis and immune-mediated acute cerebellar ataxia and myoclonus syndrome was supported by alterations in the limbic system shown in magnetic resonance imaging, lateralized discharges shown in electroencephalography, a slightly elevated protein level in the cerebrospinal fluid (CSF), and indirect immunofluorescence in the CSF with autoantibody binding to anatomical structures of the cerebellum and hippocampus. The patient improved with 2 weeks of corticosteroid treatment and four sessions of plasmapheresis. Our current case study describes a rare case of COVID-19-related limbic encephalitis with immune-mediated acute cerebellar ataxia and myoclonus syndrome (ACAM syndrome) and strengthens the need for tissue-based assays (TBAs) to screen the serum and/or CSF of patients highly suspected to have autoimmune encephalitis. We believe that the timely diagnosis and targeted aggressive immunotherapy were mainly responsible for the patient’s total recovery.

## 1. Introduction

Since the outbreak of coronavirus (COVID-19) in December 2019, accumulating evidence has supported its association with autoimmune neurological manifestations. Most of these manifestations can be life-threatening or cause severe morbidity [1]. Fortunately, most patients respond well to indicated immunomodulating therapies when they are administered in a timely way [1]. Consequently, clinicians should be particularly motivated to strive for speed and accuracy when performing differential diagnosis for neuroimmune disorders (complications) in patients with COVID-19 [2].

In general, COVID-19-related neurological manifestations are increasingly reported, either as manifestations secondary to direct viral attack on the central (CNS) or peripheral nervous system (PNS) or as a consequence of autoimmune-mediated processes [2]. The detection of viral RNA in the cerebrospinal fluid (CSF) supports the direct viral attack mechanism hypothesis, while plasma or CSF detection of specific or undetermined anti- neuronal and anti-glial antibodies supports the autoimmune mechanism hypothesis [3]. Direct COVID-19 viral attack of the CNS causes meningitis, encephalitis, vasculitis, or cerebellitis. Direct viral PNS infiltration causes hypogeusia or hyposmia [2].

Immune-mediated neurological phenomena associated with COVID-19 have been reported since the beginning of the pandemic and include peripheral diseases such as Guillain–Barré syndrome [4] and Miller Fisher syndrome variants [5]; central diseases such as limbic and brainstem encephalitis [6], acute disseminated encephalomyelitis [2,7], and acute transverse myelitis [8]; movement disorders (myoclonus or cerebellar ataxia, acute cerebellar ataxia and myoclonus (ACAM) syndrome or opsoclonus-myoclonus- ataxia syndrome (OMAS)) [5]; and muscular diseases (immune-mediated myopathy and myositis) [9].

The current case study describes a rare case of COVID-19-related limbic encephalitis with immune-mediated ACAM syndrome. We believe that the early detection of neuronal autoantibodies in the CSF with indirect immunofluorescence, which supported the autoimmune origin of the disorder, and the following aggressive immunotherapy were mainly responsible for the patient’s total recovery.

Our case presentation and updated review sheds light on the different and even unexpected clinical manifestations of COVID-19-related autoimmune encephalitis and highlights the importance of CSF evaluation in revealing the autoimmune origin of the described clinical entity, resulting in precise diagnosis and appropriate and timely treatment of the disease.

## 2. Case Presentation

A 74-year-old male patient presented in the emergency department with symptoms of confusion having started 2 days prior, left side facial numbness, and episodic arrhythmic rest tremor of the right hand. According to his relatives, in the 2 previous days, he had also expressed persistent paranoid ideation characterized by an extreme feeling of threat, as well as visual and auditory hallucinations.

During the initial neurological examination, he was found to be drowsy and disoriented, with intact language, cranial nerves, and motor and cerebellar function; mild left facial hypesthesia and some episodes of slight tremor of the right hand were observed. He had no neck rigidity.

His past medical history included cardiac surgery for mitral and tricuspid valve repair 4 years prior; he was on long-term warfarin and had a penicillin allergy.

Fourteen days before his admission, the patient was diagnosed with COVID-19 after PCR testing. He was not hospitalized because he had mild symptoms. Fatigue and olfactory dysfunction were his only described persistent symptoms. He had no prior history of COVID-19 infection or vaccination.

Chest radiography revealed reticular and hazy left lower lobe opacities. Brain computed tomography was unremarkable, depicting only minor white matter microangiopathy.

Immediately after his admission, his hallucinations became more vivid and peculiar. He described flying swords piercing his skull. He responded well to aloperidin, and the symptoms subsided.

One day later, myoclonic jerks of the four limbs, trunk, and face appeared. The intensity of myoclonic jerks gradually increased and they were exacerbated by stimulus-sensitive action myoclonus and exaggerated startle response (hyperekplexia). Simultaneously, a rapidly progressive cerebellar syndrome developed, while his consciousness remained stable without confusion or hallucinations. He was also afebrile and normotensive. Valproic acid was added to his treatment, and initial improvement in myoclonus was observed. Aloperidin administration was ceased.

Nevertheless, in the evening of the same day, he had multiple generalized convulsions of prolonged duration nonresponsive to anticonvulsive treatment. His lactic acid levels increased, and he was transferred to the intensive care unit. He was additionally treated with IV lorazepam 2 mg/day, and the seizures subsided. However, the myoclonus was unresponsive.

**Figure 1:**
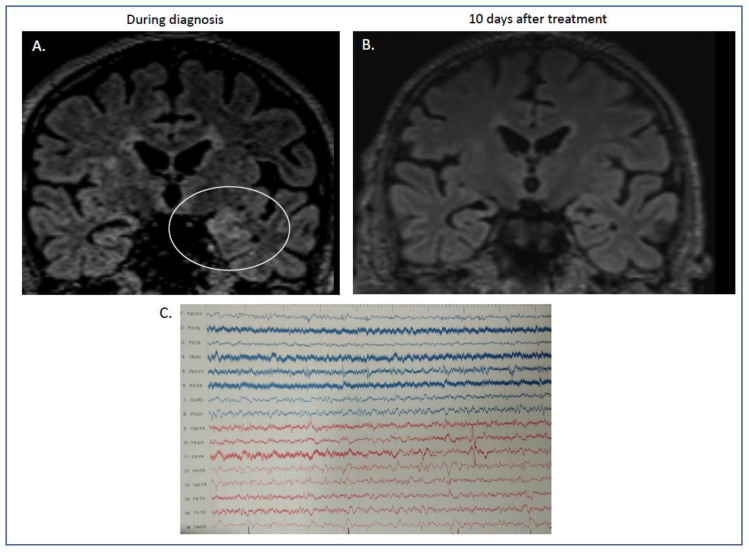
(**A**,**B**) MRI features of our case report with autoimmune limbic encephalitis after COVID-19 infection. During his stay in hospital, the initial MRI showed increased signal in the flair sequences in coronal sections of the left hippocampus ((**A**) flair, ellipse showing the maximum hyperintensity in left temporal lobe). These abnormalities were absent after immunotherapy, when our patient had recovered completely ((**B**) flair sequences). **(C**) Electroencephalogram (EEG). Electroencephalogram was performed during disease initiation. Diagram showing absence of basal rhythm with frequent recording of low-potential bradyarrhythmia, predominantly in the right hemisphere. Sparse recording of epileptiform discharges localized in the left frontotemporal areas.

At that time, RT-PCR performed with samples from the blood and tracheobronchial aspirate was positive for severe acute respiratory syndrome coronavirus-2. In contrast, the RT-PCR of the CSF was negative. Brain MRI showed Τ2 and fluid-attenuated inversion recovery with hyperintensities on the left hippocampus in coronal sections (Figure 1A).

Electroencephalography showed slow waves on the temporal derivations, mainly on the right and left posterior quadrant lateralized sparse discharges (Figure 1C).

CSF and blood samples were analyzed (Table 1). The CSF RT-PCR was negative for severe acute respiratory syndrome coronavirus-2. Serum IgG and IgM antibodies against severe acute respiratory syndrome coronavirus-2 were positive, and molecular screening for severe acute respiratory syndrome coronavirus-2 with RT-PCR with samples from the throat of the patient was also positive. Grade-3 lymphopenia was noted during the first days of hospitalization (absolute number of lymphocytes: 490, with a normal range of 1000–4800 lymphocytes/μL blood), which was resolved the subsequent days (up to 1800 cells/μL blood at the end of hospitalization).

Mild elevated protein levels (61 mg/dL), without pleocytosis (1 cell/mm^3^) and normal glucose levels 80 mg/dL) were found in the CSF analysis on the second day of hospitalization. Using routine diagnostics, the patient showed no antibodies in the serum and CSF related to known autoimmune and paraneoplastic encephalitis. In addition, no notable findings were found in terms of infectious, vascular, or systemic autoimmunity processes. All tests performed for diagnostic evaluation are shown in Table 1.

We then performed a screening assay to assess the presence of CSF or serum autoantibodies using indirect immunofluorescence on rat brain sections.

More specifically, we placed the patient’s sera (dilution 1/10) on rat brain hippocampal and cerebellar frozen tissue (Euroimmun, Lubeck, Germany; FA111m-1005-3), followed by incubation with secondary FITC-labelled anti-human IgG antibodies for visualization under a fluorescence microscope to search for any specific antibodies binding to brain tissue antigens.

**Figure 2:**
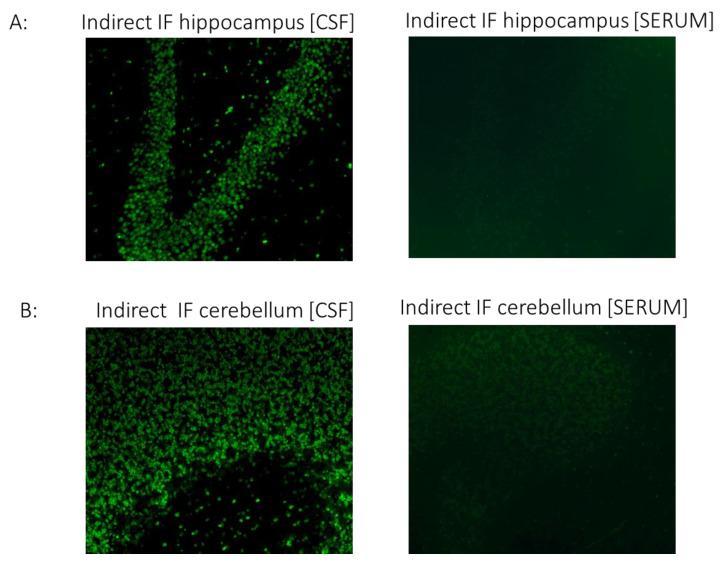
The CSF of the patient with COVID-19 shows strong IgG autoreactivity on rat brain sections, assessed using indirect immunofluorescence. Representative images of indirect immunofluorescence show autoantibody binding to circumscribed anatomical structures, including (**A**) neuronal staining in the hippocampus and (**B**) granule cells in the cerebellar granular layer. Of note, the serum sample from the patient did not exhibit significant reactivity in any brain section. COVID-19, coronavirus disease; CSF, cerebrospinal fluid; IF, indirect immunofluorescence.

CSF but not serum analysis showed strong IgG binding in brain sections. Specifically, immunostaining in cerebellar and hippocampal sections displayed a strong positive signal in the granular layers of the cerebellum and neurons of the hippocampus (Figure 2). Although the antigenic epitopes are unknown, intense staining indicates high specificity to specific neuronal proteins.

Although antigenic epitopes are currently unknown, the intense staining indicates high specificity to certain neuronal proteins.

The second brain MRI 3 days later showed persistence of the previous findings and novel bilateral brain hyperintensities on T2-weighted fluid attenuation inversion recovery on the medial temporal lobes.

As the patient fulfilled the clinical diagnostic criteria described by Graus et al. for autoimmune encephalitis [10], treatment was started with intravenous pulses of corticosteroids. Pulse steroid therapy (1000 mg intravenous methylprednisolone per day) was initiated on the third day of hospitalization, but the clinical response was insufficient after 2 days of high doses of steroids. Thus, given the severity of the symptoms and the steroid resistance, while the patient was in the intensive care unit, he was started on cycles of plasmapheresis after the second day of methylprednisolone administration. A third induction dose of 250 mg methylprednisolone was administered and subsequently quickly withdrawn. The patient underwent four plasmapheresis courses with clinical improvement and a gradual reduction in levetiracetam and valproate dosage. The results of the CSF immunostaining, suggestive of the presence of autoantibody binding to CNS structures, became available after the plasmapheresis had started and further supported the initial diagnosis. The cellular localization of the antigen is not known, but the immunostaining pattern and the response to plasmapheresis suggests a possible cell membrane antigen.

During the last days of plasmapheresis, the patient exhibited respiratory distress. Chest computed tomography showed small pleural effusions bilaterally, passive or relaxation atelectasis in the dorsal portions of the lower lobes, and ground-glass opacities in areas of both lungs, mainly in the left upper lobe, that were attributed to the emergence of transfusion-related acute lung injury (TRALI).

The patient responded immediately to plasmapheresis with a reduction in myoclonus from the first day until it resolved after the fourth day. Moreover, at the end of that week, we noticed considerable improvement in cerebellar symptoms and the restoration of swallowing capacity.

The antiepileptic treatment was further reduced, and he was discharged from the hospital on valproate 500 mg/day and oral steroid tapering (prednisone per os). One year later, he remains free of myoclonus, without cognitive deterioration, and the cerebellar ataxia has resolved. Follow-up MRI showed lesion resolution (Figure 1B).

## 3. Discussion

In this report, we have described a rare case of COVID-19-related limbic encephalitis with immune-mediated ACAM syndrome where the patient fully recovered because of timely diagnosis and proper treatment. Based on the clinical findings (new focal CNS findings, persistent cerebellar ataxia, myoclonus unresponsive to intravenous antiepileptic treatment, MRI highly suspicious for underlying encephalitis), and after excluding other possible differential diagnosis and disease mimickers, the etiology of the patient’s condition was deemed a post-infectious reaction related to COVID-19.

There are increasing reports of COVID-19-related neurological syndromes with various forms of autoimmune encephalitis (AE). The most recent systematic review on AE associated with COVID-19 [11] included 26 definite and 48 possible seronegative AE cases. Among the definite AE cases, 12 were anti-NMDAR positive, 3 were anti-GAD-65 positive (with a case of dual positivity for anti-NMDAR), 2 were anti-CASPR2 positive, 2 were myelin antibody positive, 2 were anti-GD1b positive, and 1 was positive for LGI1, GFAP, anti-GD1A, amphiphysin, and Yo.

An unidentified antibody against neuronal proteins of mouse brain structures was also reported in a case report not included in the previous review article [12], and another case of definite COVID-19-associated AE with positivity for VGKC antibodies in the serum has been reported [13].

In the review of the literature section of the last case report [13], four more cases of definite COVID-19-associated AE with positivity for myelin antibodies in the serum or CSF were reported, as well as two with unknown antineural autoantibodies directed against striatal and hippocampal neurons or the nuclei of Purkinje cells, evidenced in the CSF via immunostaining [14,15].

Later, a comprehensive review article [6] contributed more interesting cases of unidentified CSF autoantibodies resembling our case. Seven of these were originally described by Franke et al. [3], who examined the CSF of 11 critically ill COVID-19 patients with neurological manifestations. They identified anti-neuronal autoantibodies in the serum or CSF of the patients. Four of the eleven patients presented by Franke et al. [3] had identified antibodies in the serum, such as anti-NMDA and MOG. These four patients are also included as definite cases of AE associated with COVID-19 in both previously mentioned review articles [11,12,13]. Furthermore, these four patients, as well as the remaining seven examined in that study, had unknown anti-neuronal autoantibodies against the brain vessel endothelium, astrocytic proteins, and neuropil of basal ganglia, hippocampus, or olfactory bulb found via indirect immunofluorescence and cell-based assays on unfixed murine brain sections. Additionally, Franke et al. [3] stated that there is probably a causal association between the 100% presence of brain targeting autoantibodies in the CSF and the neurologic manifestations of AE, especially the clinical syndromes of hyperexcitability (myoclonus, seizures, which were present in all the patients in this survey and in our case). This encourages us to assume that CSF examination with indirect immunofluorescence could reveal similar autoantibodies in every patient with COVID-19-associated AE who presents with hyperexcitability. In another article [16] also included in the review by Ariño et al. [6], two more cases of COVID-19-associated AE with seizures and CSF immunoreactivity against brain tissues in indirect immunofluorescence were reported.

Finally, one more case of anti MOG antibodies in the serum and CSF was most recently reported by Tsouris et al. [17]. To our knowledge, 42 cases of definite COVID-19-associated AE have been reported, with 13 of them definitely diagnosed by revealing novel antineuronal autoantibodies with indirect immunofluorescence in the CSF.

The precise pathophysiological procedure of AE in COVID-19 is yet to be comprehended. Three possible mechanisms have been proposed. The most plausible is molecular mimicry in response to COVID-19, which activates host antibodies that identify self-antigens as foreign and cross-react with them, causing damage to many systems, including the CNS [18]. This mechanism is supported by the plethora of identified autoantibodies in AE cases associated with COVID-19 [11]. A classical paradigm of possible molecular mimicry as an underlying mechanism for encephalitis syndromes is cases with N-methyl-d-aspartic acid receptor (NMDAR) encephalitis after COVID-19 infection. Structural similarities of the GluN1 (synonym NR1) and GluN2a (synonym NR2a) subunits of the receptor NMDAR with the SARS-CoV-2 nonstructural protein 8 (NSP8) and 9 (NSP9) have been described. These proteins are considered important for the replication of the virus and display unique features such as direct interaction with glutamate receptors, leading to essential changes in generated action potentials and membrane resting state [19]. Vasilevska, V. et al. suggested that the pathomechanism of molecular mimicry may lead to the development of immunoglobulin of the IgG subtype against NMDAR after SARS-CoV-2 infection [20]. It is generally known that anti-NMDAR encephalitis, mediated by IgG antibodies directed against the GluN1/NR1 subunit, is characterized by the acute or subacute presentation of specific movement manifestations, encephalopathy, and psychosis-like symptoms. Pathogenetic antibodies bind to the NMDAR, leading to crosslinking of the antigen, and receptors are subsequently internalized with the result of the inhibition of the excitatory glutamatergic transmission. During acute COVID-19 infection, viral particles, including NSP8 and NSP9, are released and recognized by T cells, leading to the activation of B cells (T cell help), which mature into plasma cells producing antibodies against NSP8 and NSP9 proteins. Critical events occuring during COVID-19 infection such as SARS-CoV-2-associated endothelitis and excessive IL-17 production by activated T cells lead to the disruption of the blood–brain barrier (BBB), allowing NMDAR antibodies to enter the CNS and reach their antigen [20]. Molecular mimicry mechanisms lead to the production of antibodies produced by plasma cells able to cross-react with the NMDAR subunit GluN1 and to subsequent receptor internalization and its following degradation. Collective clinical evidence that supports the hypothesis of molecular mimicry-related mechanisms is the prolonged time interval between respiratory and neurological symptoms, instead of a concomitant involvement, as occurs during cytokine-related syndrome, and the good response to immunomodulating therapy with steroids, immunoglobulins, or plasma exchange.

The second proposed mechanism is systemic hyperinflammation secondary to hyperactivation of the immune system of the host, developing a massive release of inflammatory cytokines as a response to COVID-19 (“cytokine storm” phenomenon) [12]. These inflammatory cytokines are transported to the CNS and result in the production of encephalitis [21]. This mechanism is supported by the highly elevated inflammatory mediators in many COVID-19-related AE cases, such as IL-6 [22]. The third reported mechanism pertains to the direct invasion of severe acute respiratory syndrome coronavirus-2 into the CNS. Nevertheless, the direct invasion of the virus into the CNS is less likely to be the main process mediating COVID-19 encephalitis, as there are infrequent cases of positive CSF PCR [11]. Concurrently, there is now also accumulating evidence for associated neurologic manifestations that emerge in the immediate post-infectious phase with strong autoimmune characteristics, such as the ACAM syndrome with OMAS or without opsoclonus.

The antigenic target of our patient CSF IgG was elusive. Nevertheless, indirect evidence (clinical response to plasmapheresis, CSF, no serum binding to brain samples, and absence of underlying malignancy) suggests the presence of an antibody against an unknown extracellularly located antigen. The neuronal tissue binding observed in our patient resembles staining patterns previously described, such as in one patient with manifestations of autoimmune encephalitis presenting with malignant catatonia with COVID-19 [16]. The staining pattern in the cerebellum is also reminiscent of CSF staining patterns of cerebellum granule cells in some critically ill COVID-19 patients with rare neurological symptoms, particularly hyperexcitability (myoclonus, seizures) [3].

The autoimmune origin of this entity is supported by the time course of the disease, the positive response to immunotherapy, and the detection of neural autoantibodies in some cases. Myoclonus during the COVID-19 pandemic occurred as a post- or para-infectious immune-mediated disorder. However, we cannot entirely rule out that SARS-CoV-2 may expand transneuronally to structures connected with the olfactory bulb, as SARS-CoV-2 could affect the olfactory bulb and brainstem sequentially. Nevertheless, auotimmune manifestations with the presentation of myoclonus have been described. Franke et al., as mentioned before, described eleven critically ill COVID-19 patients presenting with unexplained neurological symptoms including myoclonus, oculomotor disturbance, delirium, dystonia, and epileptic seizures and patients analyzed for anti-neuronal and anti-glial autoantibodies in CSF and serum. Interestingly, antigenic targets included intracellular and neuronal surface proteins, such as the Yo or NMDA receptors, but, importantly, also various undetermined epitopes reminiscent of the brain tissue binding observed with certain human monoclonal SARS-CoV-2 antibodies. The potential molecular mimicry of these autoantigens of SARS-CoV-2 still awaits further verification, as well as the involved pathogenetic mechanisms (pathogenicity of antibodies or just an epiphenomenon) [3]. The production of recombinant monoclonal antibodies from the CSF (plasmablasts) of such patients and pathogenicity assessment in animal models could provide more solid information regarding their functionality. Thirty-one cases of myoclonus and ataxia associated with COVID-19 were reported in a recent review article [5], and eleven more cases were reported in five case reports and case series afterwards [23,24,25,26,27,28]. In one of these cases, anti-GFAP-antibodies were identified [29]. Data from 51 cases of patients who developed myoclonus or ataxia associated with COVID-19 revealed that the mean age of disease onset was 59.6 years (26 to 88 years), and 21.6% of these patients were female. Specific characteristics of the myoclonus were multifocal or generalized, with acute presentation, typically within 1 month of COVID-19 clinical symptoms. Myoclonus appeared in isolation (46.7%), or with ataxia (40.0%) or cognitive disturbances (30.0%). The majority of the described cases improved within 2 months under treatment with anti-epileptic medications and/or immunotherapy [5].

A definite diagnosis of classic OMAS is made when at least three of the four following characteristics are present, according to the proposed criteria [29]: (1) opsoclonus, (2) myoclonus or ataxia, (3) behavioral change or sleep disturbance, and (4) presence of tumor or anti-neuronal antibodies. Most of the COVID-19-associated cases met two of the four criteria. Thus, COVID-19-associated myoclonus and ataxia may be considered in the spectrum of OMAS. OMAS in general has been associated with viruses such as human immunodeficiency and Epstein–Barr [15] and many tumors. We can assume that the pathophysiology of COVID-19-associated myoclonus and ataxia may be similar to that of OMAS. Because OMAS responds to immunotherapy, it is considered an immune-mediated syndrome [29]. Furthermore, neuronal and cell surface antibodies have been identified in OMAS cases, including autoantibodies against Purkinje cells. These dysfunctional Purkinje cells may be responsible for the abnormal disinhibition of the deep cerebellar nuclei and, as a result, hyperexcitability of cortical and brainstem motor and non-motor areas [29]. We suppose that the same cells might be attacked by neuronal autoantibodies associated with COVID-19 in the case of COVID-19-associated AE with myoclonus and ataxia. To our knowledge, our case is the first to fulfill the diagnostic criteria described by Graus et al. for autoimmune limbic encephalitis [10] and the necessary criteria for OMAS [29].

Moreover, to our knowledge, this is the first report showing a critical adverse event (TRALI) associated with plasma exchange therapy in a patient with autoimmune encephalitis. TRALI is a distinctive form of acute respiratory distress syndrome that occurs during or within 6 h of a blood product transfusion. Clear evidence of bilateral pulmonary edema on lung imaging is necessary for the diagnosis. It is frequently misdiagnosed and fatal. It may be caused by antileukocyte antibodies in the plasma of the donor reacting with leukocyte antigens in the recipient [30,31,32]. TRALI would be the most likely cause of the acute pulmonary function deterioration in our patient because there was no sign of circulatory overload. Because of the sufficient response of the neurological syndrome to plasmapheresis and to avoid worsening of TRALI, we decided to avoid additional courses of plasmapheresis.

Overall, this case highlights the diagnostic value of CSF immunological examination in the most complicated and novel clinical entities. The present study strengthens the need for tissue-based assays (TBAs) on brain sections to screen the serum and/or CSF of patients highly suspected to have autoimmune encephalitis. Especially when TBAs are combined with cell-based assays for suspected extracellular antigen-associated encephalitis or immunoblot (or Western blot) analysis for intracellular antigen-associated encephalitis, they represent the gold standard for antigen identification.

## 4. Conclusions

This case report underlines the need for TBAs with the use of CSF (combined with serum) of patients with subacute or acute neurological manifestations suggestive of encephalitis during or after contracting COVID-19 not explained by the infection itself to further enhance the diagnostic tools for proper decision making.

The presence of hitherto-unknown neuronal autoantibodies against brain structures in the CSF of patients with COVID-19-associated AE, especially with hyperexcitability, suggests a causal pathophysiological relationship with the neurological manifestations of the infection. Exhausting immunological investigation of the CSF may be mandatory for definite diagnosis and for timely immunotherapy administration.

## Figures and Tables

**Table 1:** Diagnostic evaluation of the patient.

Immunological and COVID-Related Infection Parameters Tested			
Antibody Testing for Extracellular Synaptic Antigens			
Testing	Method	Biological Sample	Result
Anti-CASPR2	CBA	serum	negative
Anti-LGI1	CBA	serum	negative
Anti-NMDAR	CBA	CSF	negative
Anti-AMPAR1,2	CBA	serum	negative
Anti-GABAbR	CBA	serum	negative
Anti-DPPX	CBA	serum	negative
Anti-mGluR5	CBA	serum	negative
Anti-NMDAR	CBA	serum	negative
Anti-Glycin Receptor	CBA	serum	negative
Anti-AQP4	CBA	serum	negative
Anti-MOG	CBA	serum	negative
Anti-GAD65	Elisa	serum	negative
Anti-GFAP	CBA	serum	negative
Panel for intracellular antibodies			
Amphiphysin, CV2/V2/CRMP5, Hu, PNMA2, Recoverin, Ri, SOX-1, Yo, Zic4, Tr, GAD, Titin	Immunoblotting	serum	negative
Infection by SARS-CoV-2 (COVID-19)			
SARS-CoV-2 (COVID-19) RNA	RT-PCR	CSF	negative
SARS-CoV-2 (COVID-19) RNA	RT-PCR	TBS	positive
SARS-CoV-2 (COVID-19) IgM and IgG antibodies	Elisa	serum	positive
CSF analysis			
Cell count (reference control value: <5/µL)	counting (Fuchs–Rosenthal chamber)	CSF	1
Glucose (reference range: 50 to 80 mg/dL)	biochemical analyzer	CSF	80
Protein (reference range: 15 to 60 mg/dL)	biochemical analyzer	CSF	61
CSF indirect immunofluorescence (IgG)	indirect immunofluorescence on unfixed murine brain sections	serum	negative
	indirect immunofluorescence on unfixed murine brain sections	CSF	positive (granular layer of cerebellum, neuronal cells of hippocampus)

Abbreviations: COVID-19, coronavirus disease; CBA, cell-based assay; CSF, cerebrospinal fluid; TBS, tracheobronchial swab; IgG, immunoglobulin G; IgM; immunoglobulin M, mGluR5; metabotropic glutamate receptor subtype, GFAP; Glial fibrillary acidic protein, CV2/CRMP5, collapsin response mediator protein 5; CASPR2, contactin-associated protein-like 2; GABAbR, gamma-aminobutyric acid receptor B; GAD, glutamic acid decarboxylase; Zic4, Zic family member 4; GlyR, glycine receptor; MOG, myelin oligodendrocyte protein; AQP4, aquaporin-4; PNMA2, PNMA family member 2; DPPX, dipeptidyl-peptidase-like protein-6; SOX-1;,anti-Sry-like high mobility group box; AMPAR, α-amino-3-hydroxy-5-methyl-4-isoxazolepropionic acid receptor; NMDAR, N-methyl-D-aspartate receptor.

## Data Availability

All data analyzed in this study are included in the published article.
